# Intensive insulin therapy-associated costs differ substantially between ICUs

**DOI:** 10.1186/cc9814

**Published:** 2011-03-11

**Authors:** RE Harmsen, F Van Braam Houckgeest, JP Van der Sluijs, PE Spronk, MJ Schultz

**Affiliations:** 1 Academic Medical Center, Amsterdam, the Netherlands; 2 Tergooi Hospitals, Hilversum, the Netherlands; 3 Medical Center Haaglanden, The Hague, the Netherlands; 4 Gelre Hospitals, Apeldoorn, the Netherlands

## Introduction

Intensive insulin therapy (IIT) has been shown to reduce morbidity and mortality of critically ill patients [1,2]. A survey among ICU managers and nurse clinicians showed that <10% of participants evaluated costs surrounding the implementation of IIT [3]. We hypothesized IIT-associated costs to differ substantially between ICUs.

## Methods

Three ICUs developed and implemented an evidence-based guideline for IIT. For 1 year before and 1 year after implementation, all disposables and devices explicitly used for IIT were identified in each hospital. Local costs were calculated, based on costs for disposables and devices. Variable cost included costs associated with disposables. Fixed cost included costs associated with syringe pumps and point-of-care devices for blood glucose level (BGL) measurements.

## Results

A total of 2,490 patients were subjected to IIT. Patient demographics did not differ among the three ICUs and did not change over time. Median BGL declined from 119 (99 to 150) to 105 (85 to 130) mg/dl (*P *< 0.001). The number of BGL measurements per patient per day doubled from 4 (3 to 7) to 9 (5 to 12) per day (*P *< 0.001). Yearly variable costs increased from €58.574 to €118.624 (*P *< 0.001), yearly fixed costs increased from €450 to €14.282 (*P *< 0.001). Importantly, costs differed substantially from one centre to another: variable costs per patient increased from €34 (€13 to 75) to €116 (€61 to 212) (*P *< 0.001), from €13 (€5 to 44) to €48 (€32 to 88) (*P *< 0.001) and from €15 (€7 to 34) to €31 (€15 to 70) (*P *< 0.001) for the three ICUs, respectively. Fixed costs per bed per year increased from €0 to €250 (*P *< 0.001), from €13 to €384 (*P *< 0.001) and from €25 to €544 (*P *< 0.001) for the three ICUs, respectively.

## Conclusions

Glucose control-associated costs rise with the implementation of IIT. Major differences in costs are noticed when comparing ICUs with similar patient cohorts and similar blood glucose control metrics after implementation of IIT.
